# The role of acupuncture in women with advanced reproductive age undergoing in vitro fertilization-embryo transfer: A randomized controlled trial and follicular fluid metabolomics study

**DOI:** 10.1097/MD.0000000000034768

**Published:** 2023-09-08

**Authors:** Qingchang Xia, Lingyu Yu, Jingyan Song, Zhengao Sun

**Affiliations:** a College of Acupuncture and Massage, Shandong University of Traditional Chinese Medicine, Jinan, Shandong, China; b Institute of Chinese Medical Literature and Culture, Shandong University of Traditional Chinese Medicine, Jinan, Shandong, China; c The First Clinical College, Shandong University of Traditional Chinese Medicine, Jinan, Shandong, China; d Reproductive Medicine Center of Integration of Traditional and Western Medicine, Affiliated Hospital of Shandong University of Traditional Chinese Medicine, Jinan, Shandong, China.

**Keywords:** cumulative pregnancy rate, follicular fluid, kidney qi deficiency, metabolic pathways, oocyte quality

## Abstract

**Background:**

The objective of this study was to determine the efficacy of acupuncture on the outcome of in vitro fertilization (IVF) in elderly infertile patients with kidney qi deficiency, and to explore its possible mechanism from the perspective of pseudo-targeted metabolomics of follicular fluid.

**Methods:**

Sixty cases of elderly women undergoing IVF were sampled and randomly divided into 2 equal groups: the treatment and the elderly control (HA) group. In the treatment group, routine ovulation induction combined with acupuncture treatment was used. Routine ovulation induction combined with sham acupuncture was used in the HA group. Reproductive outcomes of the 2 groups were compared. The follicular fluid of patients obtained on the day of oocyte retrieval was analyzed by the ultra-high-performance liquid chromatography-mass spectrometry analysis system.

**Results:**

Compared with the HA group, the score of kidney qi deficiency syndrome in the treatment group was significantly decreased, and the 2 PN fertilization rate, high-quality embryo rate and cumulative pregnancy rate were significantly increased (*P* < .05). Through the identification of target metabolites, 3 metabolic pathways were found to be closely related to the developmental potential of oocytes, namely: Retinol metabolism pathway; Glycine, serine, and threonine metabolism pathway; Glycerophospholipid metabolism pathway.

**Conclusion:**

From our findings, acupuncture can improve the quality of oocytes thus bettering the outcome of IVF-assisted pregnancy in elderly patients with kidney qi deficiency.

**Trial registration:**

ChiCTR1800018329.

## 1. Introduction

In 2016, China implemented fully the “two-child policy,” and since then, the proportion of elderly women who want to have children has been increasing.^[1]^ The fertility of women declines as they age and after the age of 35, the decline is more significant.^[2]^ This mainly manifests by a decrease in the oocyte number and quality.^[3,4]^ As a result, more and more women with advanced reproductive age are seeking assisted reproductive technology (ART) to achieve pregnancy.

Since the birth of the first test-tube baby in 1978,^[5]^ ART has become one of the major treatment methods to solve infertility. However, its therapeutic effect on elderly patients is still unsatisfactory. Among women who undergone ART, the fertilization and transplant success rates in elderly women are lower than in young women,^[6,7]^ and the opposite is true for the miscarriage rate.^[8]^ At the same time, elderly women who received ART had a higher risk of pregnancy complications, including preeclampsia and placental abruption, compared with younger women.^[9]^ Therefore, medical research has shifted its attention to how to improve the outcome of IVF-assisted pregnancy for elderly women and explore more effective adjuvant treatment methods. Acupuncture is a unique treatment method in China with a long history. It treats systemic diseases through its unique operation method and transmission of meridional sensation. Because of the advantages such as easy operation, good curative effect, little pain, and quick effect,^[10]^ acupuncture is gradually used by scholars globally as an ART treatment method.^[11]^ However, different opinions on the efficacy of acupuncture exist with fewer studies conducted on the intervention of in vitro fertilization (IVF) for elder infertility. Besides, its mechanism of action is still elusive.

Presently, metabolomics, as a powerful analytical tool, has been applied in many fields such as biomarker discovery and traditional Chinese medicine (TCM) research, to enhance our understanding of chemical communication.^[12–15]^ Liquid chromatography-tandem mass spectrometry has become the preferred medium for metabolomics research.^[16]^ With the help of follicular fluid metabolomics, several studies have found that the amount and variety of follicular fluid metabolic components are closely related to the developmental potential and quality of oocytes.^[17–19]^ Previous studies on this topic have shown that female follicular fluid metabolites change with an increase in age.^[20]^ Additionally, the differences in follicular fluid metabolites between different age groups screened by follicular fluid metabolomics are involved in biological function, hormone synthesis and metabolism, oxidative stress, and other processes. Some of the differences also affect the outcome of IVF treatment.^[21–23]^

Based on previous findings, this study sampled elderly patients with kidney qi deficiency who planned to undergo IVF. A randomized controlled trial was adopted to conduct a scientific and standard evaluation on the outcome of elderly women IVF-assisted pregnancy with acupuncture. Targeted metabonomic analysis of follicular fluid with HPLC-MS technology is expected to provide a scientific basis for the wide application of acupuncture in ART, and new ideas and methods for the adjuvant treatment of senile infertility patients with kidney qi deficiency.

## 2. Materials and methods

### 2.1. Study design

This was a 1:1 parallel-group, randomized, single-blind (except for acupuncturists), sham-controlled trial design. The computer-generated randomized sequence was sealed in an opaque envelope. Assessors and statisticians responsible for data collection and analysis were not informed of treatment allocations.^[24]^ It was approved by the Ethics Committee of Reproduction and Genetics Center of the Affiliated Hospital of Shandong University of Chinese Medicine (No. 20180112) and prospectively registered in the Chinese Clinical Trial Registration Center (No. ChiCTR1800018329). All participants signed an informed consent when enrolled in the group. The trial was conducted and reported following the Consolidated Standards of Reporting Trials and Standards for Reporting Interventions in Clinical Trials of Acupuncture.^[25]^

### 2.2. Patients

Sixty elderly female patients, aged between 35 and 42 years old, with kidney qi deficiency who underwent IVF-assisted pregnancy, were sampled. All the patients were sampled from June 2018 to July 2019 in the Reproductive and Genetic Center of Integrated Traditional Chinese and Western Medicine of the Affiliated Hospital of Shandong University of TCM.

TCM syndrome differentiation was as follows: Its main symptom is not pregnant for a long time after marriage, menstrual color is dim, menstrual texture is thin and accompanied by lumbosacral soreness. Other symptoms included dizziness, tinnitus, decreased libido, tibial acid, knee tenderness or heel pain, and forgetfulness. The tongue was pale with a thin and white coating; the pulse was fine, especially the 2 Chi. The 3 main syndromes were required with 1 to 2 of the concurrent certificates, which could be diagnosed by combining tongue coating and pulse conditions.

Inclusion criteria were as follows: To be included in the study, the patient condition had to conform to the diagnostic criteria of infertility and the syndrome criteria of kidney qi deficiency in TCM.^[26,27]^ The body mass index (BMI) was ≤24 kg/m^2^. All the patients had a regular menstrual cycle (28–30 days) and normal ovulation. The patients had not used any steroid hormone drugs for nearly 3 months before the trial. The patients were free from any reproductive tract infection and immune disease. They had normal liver and renal function with negative hepatitis B and C virus antigens. All the patients signed informed consent.

Exclusion criteria were as follows: The patients not falling within the age range of 35 to 42, suffering from polycystic ovary syndrome (PCOS), endometriosis, hyper prolactin, uterine fibroids, and other gynecological diseases, with a history of ovarian cancer or ovarian surgery, with serious diseases, such as cardiovascular and cerebrovascular diseases, severe abnormal liver and kidney function, hematopoietic system, or psychiatric patients, with the previous history of radiotherapy and chemotherapy, who were dizzy during acupuncture and were allergic to navel therapeutic powder, who had lesions in the acupoint area and were not suitable for acupuncture, were participating in other clinical trials and those who had previously participated in the study or had undergone acupuncture as a fertility treatment were excluded from this study.

### 2.3. Clinical medication regimens

Long protocol for pituitary down-regulation of GnRH-a in luteal phase: Diphereline was administered in the middle luteal phase of the previous menstrual cycle (7 days after ovulation) by daily injection of short-acting GnRH-a of 0.1 mg or 0.05 mg until the hypophysial regulation criteria^[28]^ were met (5 mm thickness of the endometrium) under vaginal ultrasound; <5 mIU/mL concentration of both serum follicle-stimulating hormone and luteinizing hormone; E_2_ < 50 pg/mL. After 14 to 17 days of down-regulation, the gonadotropin (Gn) starting dose was determined according to the patient BMI and ovarian responsiveness. The dosage was adjusted based on blood hormone level and ultrasound monitoring follicle development. The transvaginal ultrasound was used to monitor the growth of the ovarian follicle. When the dominant follicle reached around 18 mm in diameter, and at least 3 follicles with diameter >17 mm,^[29]^ the subjects were intramuscularly injected with human chorionic gonadotropin (hCG) 8000 to 10,000 IU. About 36 hours after hCG injection, the eggs were harvested and the follicular fluid was collected and cryopreserved for later examination.

### 2.4. Intervention

The treatment was performed by acupuncturists from the Affiliated Hospital of Shandong University of TCM. The acupuncturist obtained a master degree in TCM and had at least 2 years of clinical experience.

#### 2.4.1. Acupuncture for the treatment group.

For the treatment group, acupuncture was added on the day of downregulation, 3 times a week (The frequency was determined based on the clinical experience of acupuncture experts for many years and other clinical study^[30]^). This operation was used until the day of hCG injection. The selected acupoints are Zhongwan, Guanyuan (RN4), Baihui, Yintang, bilateral Taixi, Shenshu, Sanyinjiao (SP6), Taichong, Tianshu, Dahe, Zigong (EX-CA1), Ciliao, and unilateral Zhongzhu. sterile disposable steel needles (Hwato acupuncture; 0.30 × 40 mm/0.30 × 75 mm) were inserted into the skin at a depth of 10 to 30 mm and operated manually (methods of operation include lifting, pushing and rotating) until the patient reported a needling sensation (De qi sensation). In order to maintain the “Deqi sensation,” the acupuncture technique was performed every 10 minutes during the treatment and the needle was left for 30 minutes before removal. Conversation between the acupuncturist and the patient was minimal to avoid nonspecific therapeutic effects.^[31]^

#### 2.4.2. Sham acupuncture for the HA group.

Participants in the HA group received the Streitberger placebo needle, a noninvasive placebo device, at the same acupoints.^[32]^ The needle shortens as soon as it touches the skin, giving the visual impression of being inserted into the skin. Both acupuncture and sham acupuncture used basic devices, which were difficult to distinguish from the appearance. The acupuncturist pretended to operate the needle every 10 minutes, but did not seek to “De qi.” Except that the needle was not inserted into the skin, the HA group was consistent with the treatment group in terms of acupuncture time, treatment frequency and acupoint selection.

### 2.5. Collection and preservation of follicular fluid

About 35 to 36 hours after the injection of hCG, a B-ultrasound-guided vaginal puncture was performed to extract the egg, and the first tube of bloodless pale yellow clear follicular fluid was collected. After confirming the phase of MII oocytes under the microscope, the collected follicular fluid was centrifuged at 3000g for 15 minutes. The upper fluid was aliquoted into Eppendorf tubes marked with the name of the patient and the date of egg retrieval and directly frozen in the −80°C cryogenic refrigerator for later use.

### 2.6. Outcomes measurements

The primary outcome was the cumulative pregnancy rate.

Secondary outcomes included Gn dosage, endometrial condition of HCG day, blood hormone level of HCG day, integral of kidney qi deficiency syndrome, number of oocytes retrieved, 2PN fertilization rate, 2PN cleavage rate and high-quality embryo rate.

Definition of relevant indicators as follows: The integral score of kidney qi deficiency syndrome: Patients with kidney qi deficiency have symptoms such as dizziness, tinnitus, waist, knee tenderness, etc. The severity of a single symptom was scored, and finally the score of each symptom was accumulated to obtain the kidney qi deficiency syndrome score.^[27]^ Fertilization rate of 2PN: 2PN number/obtained egg number. Cleavage rate of 2PN: 2PN cleavage number/2PN number. High-quality embryo rate: number of high-quality embryos/2PN cleavage number.^[33]^ Cumulative pregnancy rate: number of pregnancy cycles/total number of egg retrieval cycles (the pregnancy after fresh embryo or frozen embryo transplantation in this egg retrieval cycle is counted as 1 pregnancy cycle; all transplantable embryos without pregnancy are counted as 1 unpregnant cycle).

### 2.7. Experimental procedure

Follicular fluids from 62 (20 in the treatment group, 20 in the HA group, and 22 in the young control group) patients in the center of integrated traditional Chinese and western medicine reproductive and genetic center of the affiliated hospital of Shandong University of TCM was used. The baseline data of the 3 groups were balanced and comparable except for age and basic endocrine (Table 1). The specific experimental procedure is described below.

**Table 1:** The basic data of patients comparison among the 3 groups ($\bar{x}\pm s$, %).

Base data	Treatment group (n = 30)	HA group (n = 30)	Control group (n = 30)	*P* value
Age (yr)	37.70 ± 2.81	37.27 ± 1.95	29.83 ± 4.35	<.001^b,c,^**
History of gestation	20.0% (6/30)	23.3% (7/30)	13.3% (4/30)	.602
Years of infertility (yr)	3.50 ± 2.42	3.70 ± 2.65	3.50 ± 1.59	.085
BMI (kg/m^2^)	22.50 ± 1.59	22.81 ± 1.39	22.12 ± 1.47	.543
Male factors	16.7% (5/30)	23.3% (7/30)	20.0% (6/30)	.812
bFSH (mIU/mL)	14.27 ± 3.83	14.60 ± 3.05	6.85 ± 1.44	<.001^b,c,^**
bLH (mIU/mL)	4.55 ± 1.85	4.43 ± 1.74	4.68 ± 1.89	.838
bE_2_ (pg/mL)	45.19 ± 5.75	45.27 ± 6.24	36.52 ± 10.22	<.001^b,c,^**
bAFC (n)	11.00 ± 3.56	10.93 ± 3.31	17.97 ± 6.29	<.001^b,c,^**
Whole embryo freezing strategy	20.0% (6/30)	26.7% (8/30)	23.3% (7/30)	.830

“a” was statistically significant for the treatment group and the HA group, “b” was statistically significant for the treatment group and the control group, and “c” was statistically significant for the HA group and the control groups.

HA = elderly control.

*P < .05.

** *P* < .01.

#### 2.7.1. Sample preparation.

The frozen follicular fluids first were melted at 4°C. Accurately measured 100 µL was then transferred into a 1.5 mL centrifuge tube. A total of 300 µL methanol, internal standard 500 ng/mL chloramphenicol, and control were added and then vortexed for 5 minutes. The tube with the content was centrifuged at 14,000g high-speed at 4°C for 30 minutes. The supernatant was absorbed into the EP tube and 10 µL samples were taken for analysis.

#### 2.7.2. Chromatographic condition.

The Waters HILIC column (100*2.1 mm, 1.7 µm) was used. For the positive ion mode, mobile phase A was aqueous containing 10 mL ammonium acetate. Mobile phase B was an acetonitrile solution containing 0.1% formic acid in the organic phase. The flow rate was set at 0.4 mL/min and the column temperature was set at 40°C.^[16]^

#### 2.7.3. Mass spectrometry conditions.

Data were collected in positive and negative ion modes by MASS spectrometry using AB SCIEX API 4000QTRAP.^[34]^ The scanning method used was classic multiple reaction monitoring (MRM), with 35 psi air curtain gas, 55 psi atomization gas, 55 psi auxiliary atomization gas, ion source temperature of 550°C, 100 V cluster removal voltage, and a residence time of 1 ms.

#### 2.7.4. Differential metabolite screening.

The PCA and PLS-DA methods of MetaboAnalyst analysis software were used to analyze the samples visually, reliably, and for statistical significance on the whole. Each point on the score plot represented a corresponding sample, and the separation between groups of samples could be seen. The VIP value represented the contribution of each measured variable to the separation between samples. The larger the VIP value, the greater the contribution. Generally, a significant difference was considered only for the VIP values >1. At the same time, a *t* test was used for statistical analysis, and a *P* value <.05 indicated a significant difference.

#### 2.7.5. Metabolic pathway analysis (MetPA).

Metabolic pathway analysis database identified possible biodisturbed metabolic pathways through metabolic pathway concentration and topological analysis. It also analyzed the relevant metabolic pathways of different metabolites in the 3 groups with the hypergeometric test data analysis algorithm and Relative-Betweenness Centrality topology structure. Based on the relative response values of metabolites identified in the metabolic pathways and the dimensionality reduction algorithm, the relative response values of the metabolic pathways were obtained. The correlation coefficients between the metabolic pathways were calculated to draw the metabolic pathway association network diagram.

### 2.8. Statistical analysis of clinical data

SPSS 22.0 statistical software was used for analysis. The mean ± standard deviation ($\bar{X}\pm S$) was used for the statistical description of the measurement data of each group conforming to the normal distribution. For the measurement data not conforming to the normal distribution, the median and quartile were used for statistical description. Analysis of variance and rank-sum test were used to compare the changes in values that accord with normal and nonnormal distribution, respectively. An independent sample *t* test was used for comparison between the 2 groups. The data were statistically described by frequency (composition ratio) and Pearson chi-square test. A *P* < .05 was considered statistically significant.

## 3. Results

### 3.1. Clinical outcomes

#### 3.1.1. Comparison of basic data between the treatment group and the HA group.

As shown in Table 2, there were no statistically significant differences in age, history of gestation, infertility years, BMI, male factor, bFSH, bLH, bE_2_, bAFC, and whole-embryo freezing strategy between the treatment group and the HA group (*P* > .05).

**Table 2:** Comparison of baseline data between the treatment group and the HA group ($\bar{x}\pm s$, %).

Base data	Treatment group (n = 30)	HA group (n = 30)	*P* value
Age (yr)	37.70 ± 2.81	37.27 ± 1.95	.490
History of gestation	20.0% (6/30)	23.3% (7/30)	.754
Years of infertility (yr)	3.50 ± 2.42	3.70 ± 2.65	.761
BMI (kg/m^2^)	22.50 ± 1.59	22.81 ± 1.39	.420
Male factors	16.7% (5/30)	23.3% (7/30)	.519
bFSH (mIU/mL)	14.27 ± 3.83	14.60 ± 3.05	.705
bLH (mIU/mL)	4.55 ± 1.85	4.43 ± 1.74	.802
bE_2_ (pg/mL)	45.20 ± 5.75	45.27 ± 6.24	.964
bAFC (n)	11.00 ± 3.56	10.93 ± 3.31	.940
Whole embryo freezing strategy	20.0% (6/30)	26.7% (8/30)	.542

HA = elderly control.

#### 3.1.2. Comparison of clinical data between the treatment group and the HA group.

Clinical data of the groups of study are presented in Table 3. A significant difference between the 2 groups was noticed in the fertilization rate of 2PN, high-quality embryo rate, cumulative pregnancy rate, and the integral score of kidney qi deficiency syndrome (*P* < .05). No significant difference was observed in Gn dosage, E_2_ at hCG day, endometrium thickness at hCG day, type A endometrium, the number of oocyte retrieval, and cleavage rate of 2PN between the treatment and the HA groups (*P* > .05).

**Table 3:** Comparison of clinical data between the treatment group and the HA group ($\bar{x}\pm s$, %).

Clinical data	Treatment group (n = 30)	HA group (n = 30)	*P* value
Gn days	11.67 ± 1.49	11.53 ± 2.26	.788
Gn dosage	3231.67 ± 810.57	3340.00 ± 871.35	.620
E_2_ at HCG day (pg/mL)	2674.83 ± 1326.07	3329.90 ± 1463.37	.074
Em at HCG day (cm)	1.09 ± 0.28	1.20 ± 0.20	.088
Type A endometrium	63.3% (19/30)	60.0% (18/30)	.791
The number of oocyte retrieval	9.27 ± 4.02	7.40 ± 3.43	.058
Fertilization rate of 2PN	61.9% (172/278)	46.4% (103/222)	.001**
Cleavage rate of 2PN	86.0% (148/172)	83.5% (86/103)	.565
High-quality embryo rate	61.5% (91/148)	44.2% (38/86)	.010*
Cumulative pregnancy rate	50.0% (14/28)	18.2% (4/22)	.042*
Integral score of kidney qi deficiency syndrome (down-regulation day-HCG day)	5.63 ± 2.16	−0.10 ± 1.06	<.001**

HA = elderly control.

* *P* < .05.

** *P* < .01.

### 3.2. Experimental results

#### 3.2.1. Metabolite hierarchy clustering analysis heat map.

**Figure 1.**
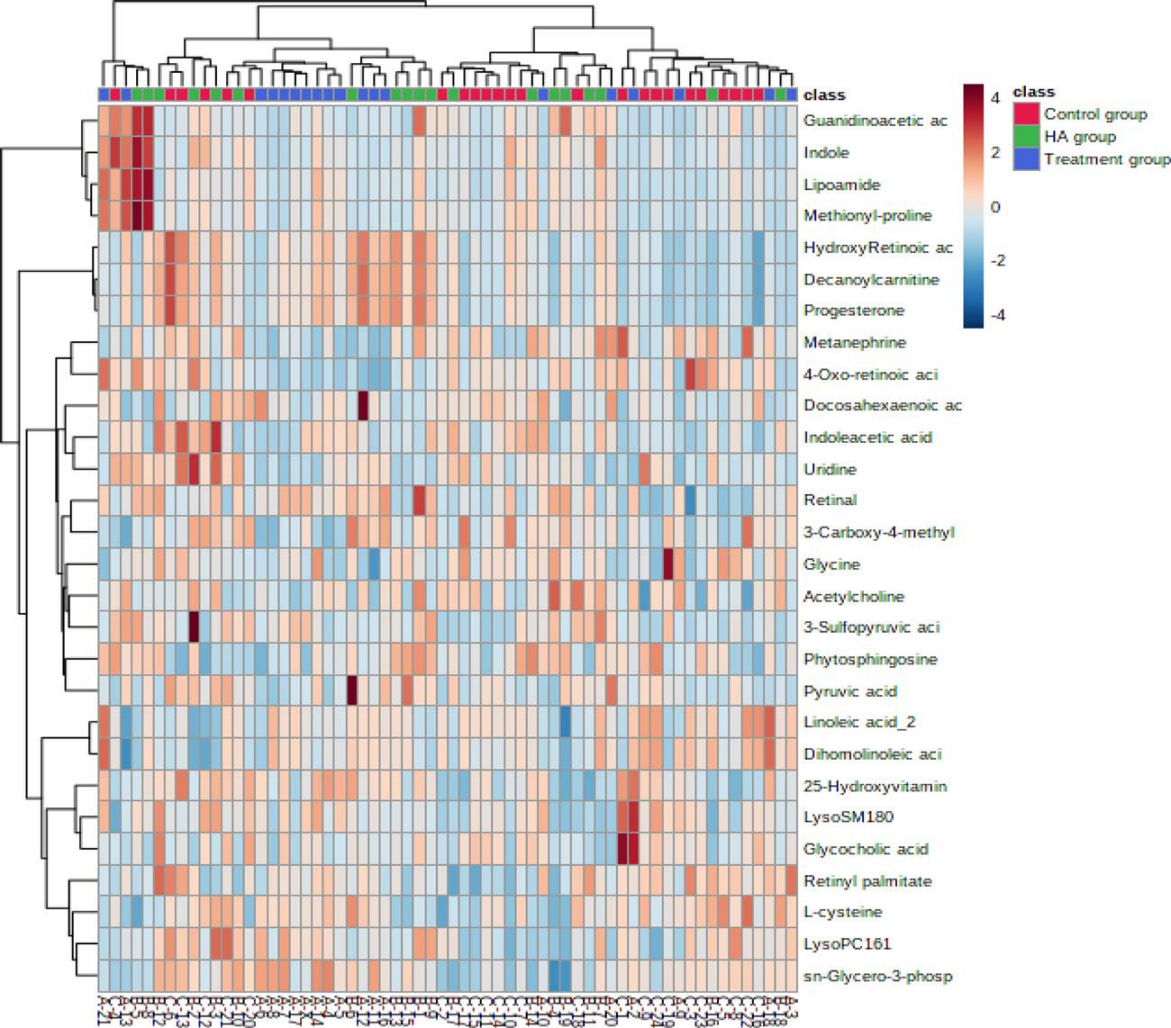
Heat maps of related metabolites of treatment group, HA group and control group. HA = elderly control.

As seen in the heatmap (Fig. 1), the sample and metabolites differences were simultaneously hierarchically clustered. The horizontal axis of the figure shows a dendrogram of the samples. The samples of the HA group and the treatment group were clustered together. The concentrations of different metabolites varied significantly between the HA group and the treatment group. The vertical axis of the figure shows a dendrogram of the metabolite differences. Obvious clustering and relevance of the metabolite differences are shown in the hierarchical cluster analysis. The results of hierarchical cluster analysis provided a distinct visualization of the groups. The metabolites in the same or similar metabolic pathways were clustered together.

#### 3.2.2. PLS-DA scores plot.

**Figure 2.**
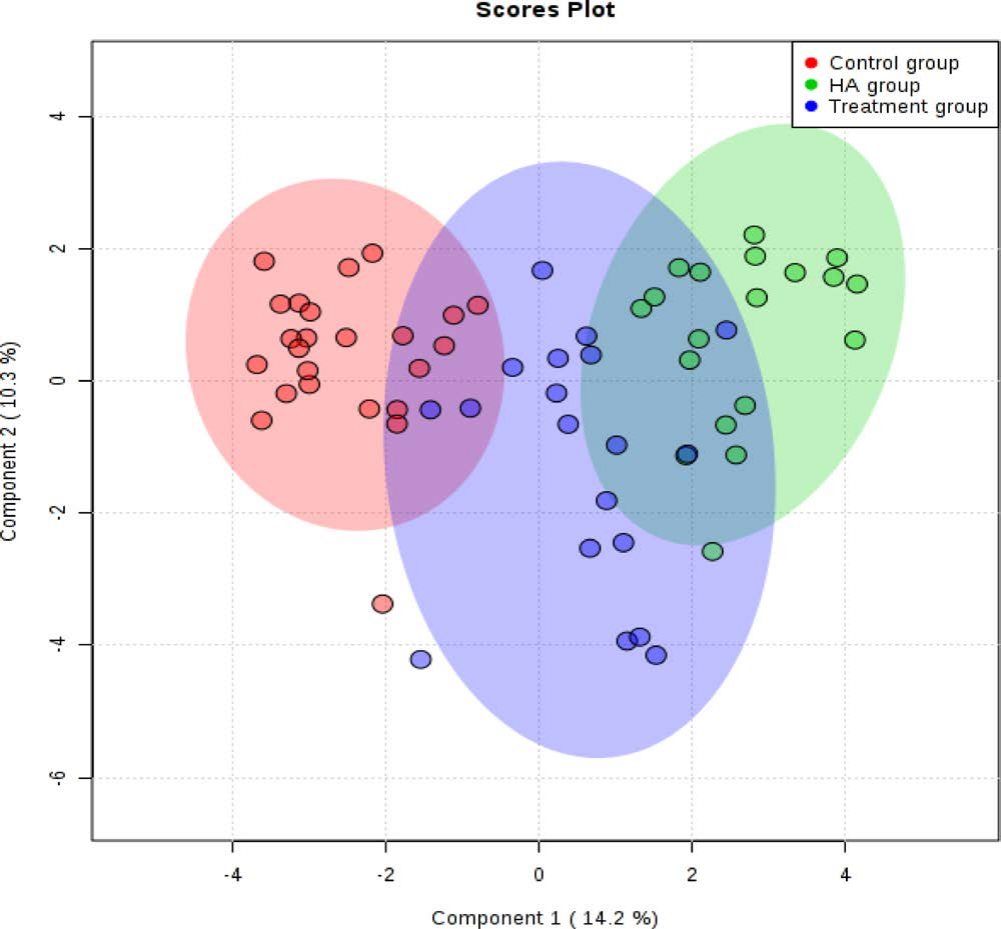
PLS-DA scores plot of treatment group, HA group and control group. HA = elderly control.

The HA group and the control (youth control) group were well separated, and the aggregation was good in the groups (Fig. 2). After the acupuncture intervention, the PLS-DA score plot of the treatment group got generally closer to the control group.

#### 3.2.3. The VIP score diagram and box diagram of different metabolites.

**Figure 3.**
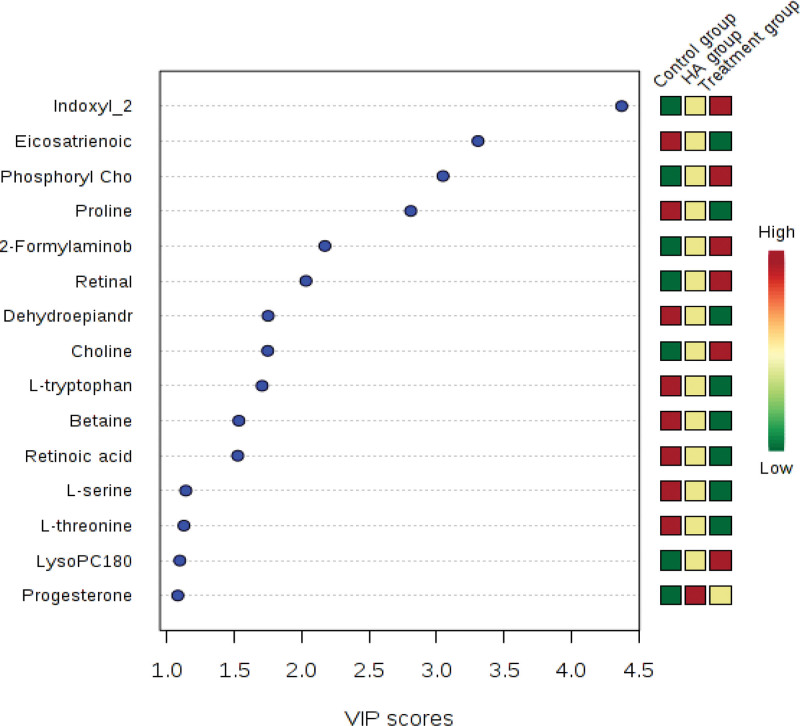
The VIP score diagram of treatment group, HA group and control group. HA = elderly control.

**Figure 4.**
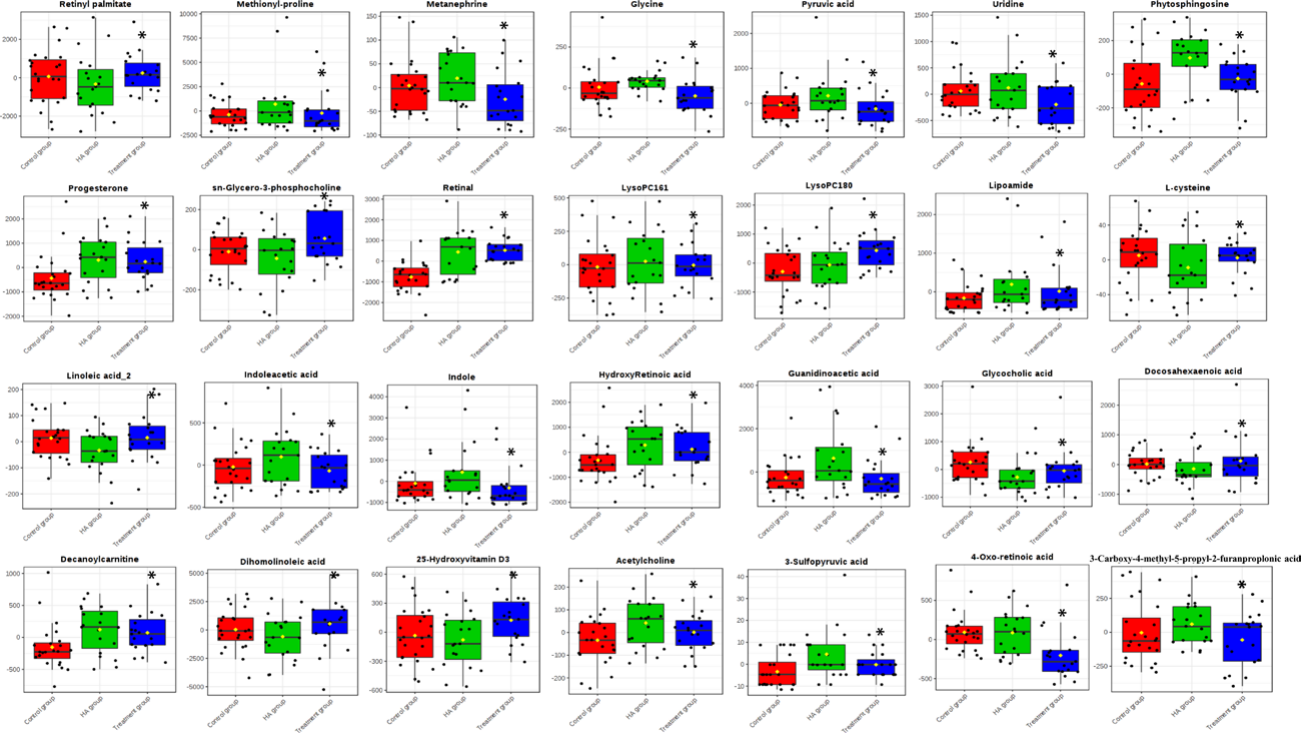
Box diagrams of different metabolites in treatment group, HA group and control group. HA = elderly control.

The samples were well separated between the groups (Fig. 3). Compared with the HA group, 28 substances in the treatment group were either up-regulated or down-regulated (Fig. 4). Nine metabolites were up-regulated: Retinyl palmitate, Sn-Glycero-3-phosphocholine, LysoSM (18:0), L-cysteine, Linoleic acid, Glycocholic acid, Docosahexaenoic acid, Dihomolinoleic acid, and 25-hydroxyvitamin D3. A total of 19 substances were down-regulated, including 3-Sulfopyruvic acid, 4-Oxo-retinoic acid and 3-Carboxy-4-methyl-5-propyl, Methionyl-proline, Metanephrine, Glycine, Pyruvic acid, Uridine, Phytosphingosine, Progesterone, Retinol, LysoPC (16:1), Lipoamide, Indoleacetic acid, Indole, Hydroxy Retinoic acid, Guanidinoacetic acid, Decanoylcarnitine, Acetylcholine, 3-Sulfopyruvic acid, 4-Oxo-retinoic acid and 3-Carboxy-4-methyl-5-propyl.

#### 3.2.4. Metabolic pathway analysis diagram.

**Figure 5.**
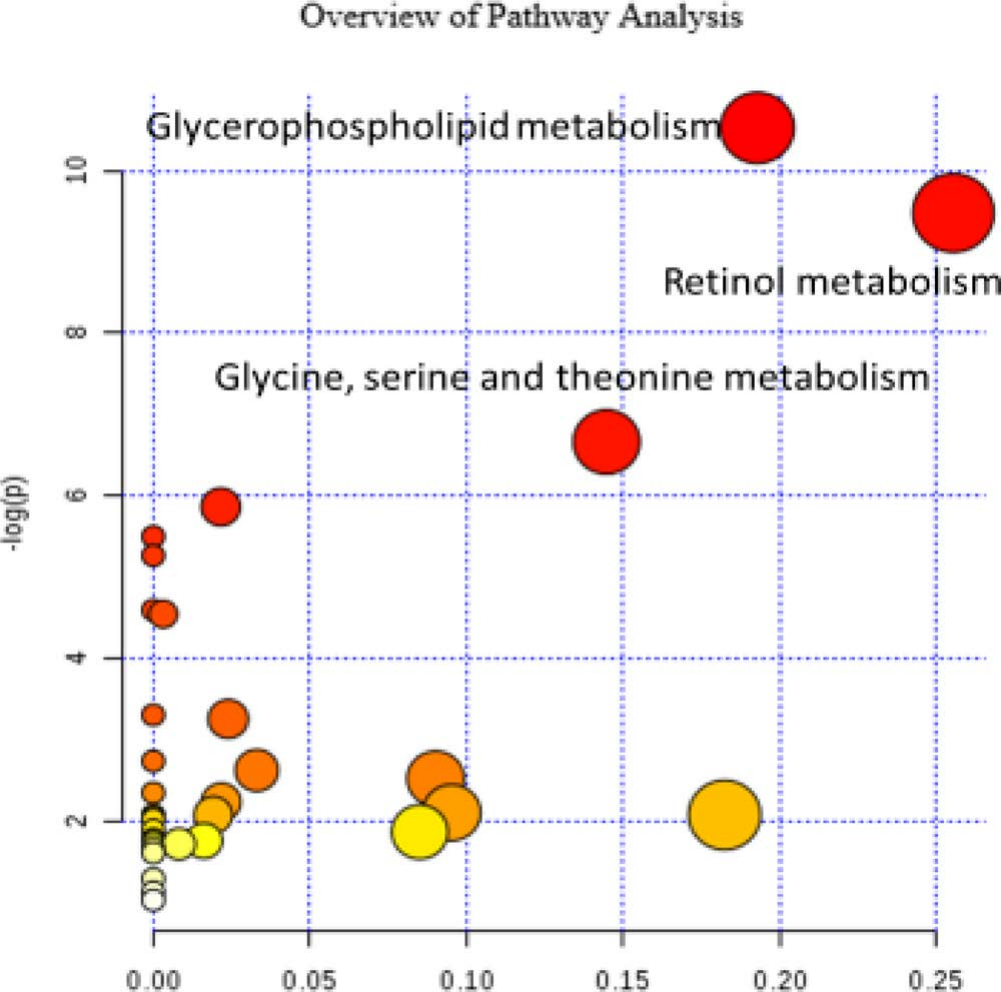
Metabolic pathway analysis diagram of treatment, HA and control groups. HA = elderly control.

Through the pathway analysis of different metabolites (Fig. 5), 3 major related metabolic pathways were found, namely, Retinol metabolism, Glycine, Serine and threonine metabolism, and Glycerophospholipid metabolism.

## 4. Discussion

In this study, we determined the effect of acupuncture on the outcome of IVF in elderly infertile women with kidney qi deficiency and explored its possible mechanism from the perspective of pseudo-targeted metabolomics of follicular fluid. We found that the 2PN fertilization rate, high-quality embryo rate, and cumulative pregnancy rate of the treatment group were significantly increased compared with the HA group (*P* < .05). At the same time, the score, and the symptoms of kidney qi deficiency syndrome of the treatment group were significantly decreased and improved after acupuncture intervention, respectively. As can be seen from (Fig. 2), The HA and the control groups were well separated, and the aggregation was good in the groups as evidenced by the PLS-DA scores plot. After the acupuncture intervention, the PLS-DA scores plot of the treatment group was close to the control group, indicating that acupuncture had a certain therapeutic effect. The improvement of the treatment group results mainly benefited from the therapeutic effect of acupuncture, choosing Zhongwan, RN4, Tianshu, Dahe, EX-CA1, and Ciliao for acupuncture can regulate chongren while stimulating local uterine blood circulation and improving uterine blood supply. Baihui and Yintang can calm the nerves and relieve patients’ anxiety. Selecting Taixi, Shenshu, SP6, and Taichong for acupuncture can soothe the liver, strengthen the spleen and tonify the kidney. Acupuncture Zhongzhu can regulate triple energizer.

Previous study on non-targeted metabolomics screened out several metabolic markers in follicular fluid of elderly patients with kidney qi deficiency.^[22]^ The present study verified these metabolic markers through pseudo-targeted metabolomics. The main metabolic pathways were also analyzed and discussed. From the analysis of the follicular fluid of the 3 groups, 28 substances were found to be either up-regulated or down-regulated in the treatment group compared with the HA group. In this study, it was found that after acupuncture intervention, the content of L-Cysteine with antioxidant properties and the rate of 2PN fertilization were increased in follicular fluid, which may be related to changing the oxidative stress of oocytes. Previous study have found that the scavenging efficiency of reactive oxygen species in follicular fluid of elderly women was significantly reduced,^[35]^ When the balance of follicular fluid oxygenation was maintained, DNA damage and apoptosis were reduced, and the quality of oocytes in older women was improved,^[36,37]^ It is consistent with our view. At the same time, previous studies have shown that acupuncture can improve ovarian reserve function and oocyte quality,^[38,39]^ the level of insulin-like growth factor and serum β-endorphin of follicular fluid were significantly increased by acupuncture at RN4, SP6, EX-CA1, and Zhongji (CV3) point, and the rate of high-quality eggs and embryos was improved, and the symptoms of kidney deficiency were relieved.

Through the pathway analysis of different metabolites, there were 3 major related metabolic pathways, namely Retinol metabolism, Glycine, serine and threonine metabolism, and Glycerophospholipid metabolism. The 3 metabolic pathways are described below respectively.

### 4.1. Retinol metabolism

The main function of retinol is cell signal transduction through binding to the 2 types of retinol receptors in the nuclear hormone receptor family, RARs, and RXRs. These receptors are transcription factors regulated by ligands and are essential for embryonic development and adult physiology. It has been found that the embryo quality of adult oocytes was improved after the addition of retinol.^[40]^ The biological importance of retinol has long been known since major developmental abnormalities occur after retinol deprivation or exposure to excess retinol. Some of these abnormalities resemble congenital birth defects, which are more common in humans, including spina bifida and cleft palate.^[41]^ Hydroxy Retinoic acid, also known as tretinoin, is an intermediate product of retinol metabolism in the body, with a similar effect as retinol.^[42]^ 4-oxyretinoic acid, an inactivated pathway in retinoic acid growth and differentiation, has been alleged to be a highly active metabolite that can regulate the position of early embryo.^[43]^ After acupuncture intervention, retinol content was down-regulated while retinol palmitate was up-regulated in the treatment group compared with the HA group. It could be that the intervention of acupuncture allowed for the degradation of retinol into retinol palmitate which is more easily absorbed.

As for the synthesis and decomposition of retinol, it has been reported that retinol is transformed into retinaldehyde through 2 oxidation steps, and then into all-trans retinoic acid. All-trans retinoic acid is present in high levels in embryonic and adult tissues and effectively binds to RARs. It is the main bioactive retinoic acid in vivo and could be having important biological functions.^[44,45]^ In this study, Retinyl Palmitate was first metabolized to Retinal, and then to Hydroxyretinoic acid. In the HA group, the content of Retinyl Palmitate decreased, while that of Retinal and Hydroxyretinoic acid increased. It is speculated that the activity of metabolic enzymes that catalyze the production of Retinal from Retinyl palmitate was increased in the HA group, leading to an increase in the production of Retinal. This further increased the content of downstream Hydroxyretinoic acid. After acupuncture treatment, the activity of this abnormal metabolic enzyme was regulated, resulting in the up-regulation of Retinyl Palmitate in the treatment group and reduction in the content of Retinal and Hydroxyretinoic acid close to that level in the control group.

### 4.2. Glycine, serine, and threonine metabolism

Glycine, serine, and threonine are closely related to the growth of microorganisms,^[46]^ and their content is directly related to the normal growth of microorganisms. Glycine transformation not only affects the clinical pregnancy and live birth rate^[47]^ but is also related to the normal chromosomes of human embryos during the cleavage stage.^[48]^ A previous study^[49]^ found that when cutoff values for the conversion of alanine, arginine, glutamine, leucine, and tryptophan were selected and modeled to predict fertilization and cleavage potential, oocytes that did not exceed cutoff values of ≥2 of these key amino acids were more likely to lyse. The data indicated that noninvasive amino acid spectrum analysis could be used to measure oocyte development. Another study^[50]^ found that navel therapy could regulate glycine content in the body, and then affect the body neural, endocrine, and immune networks.

In this metabolism pathway, Glycine, serine, and threonine were metabolized into Guanidinoacetic acid, Pyruvic acid, and L-cysteine respectively. In the HA group, the contents of Pyruvic acid and L-cysteine were upregulated and increased, while the contents of Guanidinoacetic acid were reduced. It was speculated that the activity of metabolizing Glycine into Guanidinoacetic acid was weakened, leading to the reduced production of Guanidinoacetic acid thus Glycine accumulated and increase in content. Xia et al^[51]^ found that the follicular fluid of patients with repeated failures of IVF had several amino acids (valine, threonine, isoleucine, cysteine, serine, proline, alanine, phenylalanine, lysine, methionine, and ornithine) compared with those of normal fertilized patients. Our study confirmed that the increase of threonine, serine, and cysteine might cause the quality of oocytes to decrease, and it is not conducive to later perform IVF. We found that after acupuncture intervention, the activity of metabolic enzymes in the Guanidinoacetic acid was recovered to a certain extent, the Glycine metabolism into Guanidinoacetic acid increased, while the content of Glycine decreased. This further resulted in the decrease of the other 2 metabolites, Pyruvic acid, and L-cysteine, which were close to the level of the control group.

### 4.3. Glycerophospholipid metabolism

One study^[52]^ conducted an untargeted metabolomic analysis of follicular fluid in PCOS patients and normal patients. It found that reduced phosphatidyl glycerophosphate and triglyceride levels were highly correlated with reduced PCOS fertilization rate. Metabolic dysfunction in the biosynthesis of glycerophospholipids and sphingolipids in follicles can reduce 2PN fertilization in PCOS patients during IVF. In the present study, LysoPC (16:1) was metabolized into Sn-glycero-3-phosphocholine in the Glycerophospholipid metabolism pathway. LysoPC (16:1) was increased in the HA group while Sn-glycero-3-phosphocholine was decreased. It was speculated that LysoPC (16:1) metabolism into Sn-glycero-3-phosphocholine was reduced, resulting in decreased production of Sn-glycero-3-phosphocholine while LysoPC (16:1) accumulation was increased. After acupuncture intervention, LysoPC (16:1) metabolism into Sn-glycero-3-phosphocholine was restored to a certain extent, resulting in an increase in the production of Sn-glycero-3-phosphocholine and a decrease in LysoPC (16:1) level close to that of the control group.

This study was a confirmatory study of follicular fluid metabolites and their pathways in elderly patients with kidney qi deficiency. The standard ovulation induction regimen was chosen as the GnRH-a luteal phase long protocol. Although the difference in the regimens was avoided and thus, did not affect the clinical results, the application results of acupuncture in other ovulation induction regimens still need to be further studies. Due to the high cost of sample testing, the sample size of this study was relatively small. A multi-center, large-sample randomized controlled trial should be carried out to further confirm our findings.

## 5. Conclusions

We used pseudo-targeted metabolomics to identify the different metabolites in the follicular fluid of the 3 groups of patients. From our results, 28 metabolites involving 3 metabolic pathways that are mainly related to follicular quality are either up-regulated or down-regulated after acupuncture intervention. These 3 pathways are retinol metabolism, glycine, serine, and threonine metabolism, and glycerophospholipid metabolism pathways. Acupuncture improves the syndrome of kidney qi deficiency in elderly patients through the regulation of the above metabolic pathways thus, improving the fertilization rate of 2PN, the high-quality embryo rate, and the cumulative pregnancy rate.

## Acknowledgments

We acknowledge the contributions of the co-researchers, field workers and tutors. We also thank the participants and their family members for their cooperation throughout the recruitment and data collection processes.

## Author contributions

**Data curation:** Jing-Yan Song.

**Formal analysis:** Jing-Yan Song.

**Investigation:** Lingyu Yu.

**Methodology:** Jing-Yan Song.

**Supervision:** Jing-Yan Song.

**Visualization:** Zhengao Sun.

**Writing – original draft:** Qingchang Xia.

**Writing – review & editing:** Zhengao Sun.

## References

[1] Teng X Shane M Pan S. The changing situation about maternal age, risk factors and pregnancy outcomes after the two-child policy: a retrospective cohort study. Ann Palliat Med. 2020;9:824–34. 32312075 10.21037/apm.2020.04.27

[2] Correa-de-Araujo R Yoon S. Clinical outcomes in high-risk pregnancies due to advanced maternal age. J Womens Health (Larchmt). 2021;30:160–7. 33185505 10.1089/jwh.2020.8860 PMC8020515

[3] Li C Lin L Tsai H. The molecular regulation in the pathophysiology in ovarian aging. Aging Dis. 2021;12:934–49. 34094652 10.14336/AD.2020.1113 PMC8139203

[4] Babayev E Duncan F. Age-associated changes in cumulus cells and follicular fluid: the local oocyte microenvironment as a determinant of gamete quality. Biol Reprod. 2022;106:351–65. 34982142 10.1093/biolre/ioab241 PMC8862720

[5] Katler Q Kawwass J Hurst B. Vanquishing multiple pregnancy in in vitro fertilization in the United States-a 25-year endeavor. Am J Obstet Gynecol. 2022;227:129–35. 35150636 10.1016/j.ajog.2022.02.005

[6] Dal Canto M Bartolacci A Turchi D. Faster fertilization and cleavage kinetics reflect competence to achieve a live birth after intracytoplasmic sperm injection, but this association fades with maternal age. Fertil Steril. 2021;115:665–72. 32888678 10.1016/j.fertnstert.2020.06.023

[7] Kato K Ueno S Berntsen J. Comparing prediction of ongoing pregnancy and live birth outcomes in patients with advanced and younger maternal age patients using KIDScore™ day 5: a large-cohort retrospective study with single vitrified-warmed blastocyst transfer. Reprod Biol Endocrinol. 2021;19:98. 34215265 10.1186/s12958-021-00767-4 PMC8252298

[8] Cabry R Merviel P Hazout A. Management of infertility in women over 40. Maturitas. 2014;78:17–21. 24679892 10.1016/j.maturitas.2014.02.014

[9] Wu Y Chen Y Shen M. Adverse maternal and neonatal outcomes among singleton pregnancies in women of very advanced maternal age: a retrospective cohort study. BMC Pregnancy Childbirth. 2019;19:3. 30606150 10.1186/s12884-018-2147-9 PMC6318893

[10] Xia Q Sun Z Song J. Research progress of acupuncture and moxibustion in in vitro fertilization-embryo transfer. Chin J Reprod Contracept. 2018;38:784–7.

[11] Çoksüer H Barut M Bozkurt M. Acupuncture enhances chances of pregnancy in unexplained infertile patients who undergo a blastocyst transfer in a fresh-cycle. Chin J Integr Med. 2019;25:298–302. 31236890 10.1007/s11655-018-2918-6

[12] Zhang A Liu Q Zhao H. Phenotypic characterization of nanshi oral liquid alters metabolic signatures during disease prevention. Sci Rep. 2016;6:19333. 26785698 10.1038/srep19333 PMC4726315

[13] Qu X Gao H Sun J. Identification of key metabolites during cisplatin-induced acute kidney injury using an HPLC-TOF/MS-based non-targeted urine and kidney metabolomics approach in rats. Toxicology. 2020;431:152366. 31926187 10.1016/j.tox.2020.152366

[14] Wu H Wang L Zhan X. A UPLC-Q-TOF/MS-based plasma metabolomics approach reveals the mechanism of Compound Kushen Injection-based intervention against non-small cell lung cancer in Lewis tumor-bearing mice. Phytomedicine. 2020;76:153259. 32534358 10.1016/j.phymed.2020.153259

[15] Brunetti A Carnevale Neto F Vera M. An integrative omics perspective for the analysis of chemical signals in ecological interactions. Chem Soc Rev. 2018;47:1574–91. 29114668 10.1039/c7cs00368d

[16] Nan Y Zhou X Liu Q. Serum metabolomics strategy for understanding pharmacological effects of ShenQi pill acting on kidney yang deficiency syndrome. J Chromatogr B Analyt Technol Biomed Life Sci. 2016;1026:217–26. 10.1016/j.jchromb.2015.12.004 26747643

[17] Zhang Y Zhu Z Li H. Resolvin E1 in follicular fluid acts as a potential biomarker and improves oocyte developmental competence by optimizing cumulus cells. Front Endocrinol. 2020;11:210. 10.3389/fendo.2020.00210 PMC7176900 32373069

[18] Takahashi N Harada M Azhary J. Accumulation of advanced glycation end products in follicles is associated with poor oocyte developmental competence. Mol Hum Reprod. 2019;25:684–94. 31504800 10.1093/molehr/gaz050

[19] Huang P Zhou Y Tang W. Long-term treatment of Nicotinamide mononucleotide improved age-related diminished ovary reserve through enhancing the mitophagy level of granulosa cells in mice. J Nutr Biochem. 2022;101:108911. 34801690 10.1016/j.jnutbio.2021.108911

[20] Dogan B Karaer A Tuncay G. High-resolution H-NMR spectroscopy indicates variations in metabolomics profile of follicular fluid from women with advanced maternal age. J Assist Reprod Genet. 2020;37:321–30. 31942667 10.1007/s10815-020-01693-x PMC7056815

[21] Zhang X. Preliminary screening of biomarkers for elderly IVF/ICSI patients with renal qi deficiency based on follicular fluid metabolomics. Master. Shandong University of Traditional Chinese Medicine; 2017.

[22] Zhang X Wang T Song J. Study on follicular fluid metabolomics components at different ages based on lipid metabolism. Reprod Biol Endocrinol. 2020;18:42. 32398082 10.1186/s12958-020-00599-8 PMC7216654

[23] Zhang X Yu Y Sun Z. Metabolomic analysis of human follicular fluid: potential follicular fluid markers of reproductive aging. J Pak Med Assoc. 2018;68:1769–81. 30504940

[24] Qin Z Ding Y Xu C. Acupuncture vs noninsertive sham acupuncture in aging patients with degenerative lumbar spinal stenosis: a randomized controlled trial. Am J Med. 2020;133:500–507.e520. 31525334 10.1016/j.amjmed.2019.08.038

[25] Zhang N Tu J Lin Y. Overall reporting descriptions of acupuncture for chronic pain in randomized controlled trials in English journals. J Pain Res. 2021;14:2369–79. 34393507 10.2147/JPR.S319195 PMC8354735

[26] Carson S Kallen A. Diagnosis and management of infertility: a review. JAMA. 2021;326:65–76. 34228062 10.1001/jama.2021.4788 PMC9302705

[27] Yan S Zhang L Wang M. Study on operation standard of grade evaluation of syndrome differentiation factor of kidney deficiency syndrome. J Chengdu Univ Chin Med. 2001;01:56–9.

[28] Song J Duan C Cai W. Comparison of GnRH-a prolonged protocol and short GnRH-a long protocol in patients with thin endometrium for assisted reproduction: a retrospective cohort study. Drug Des Devel Ther. 2020;14:3673–82. 10.2147/DDDT.S270519 PMC7505707 32982174

[29] So E Ng E Wong Y. A randomized double blind comparison of real and placebo acupuncture in IVF treatment. Hum Reprod. 2009;24:341–8. 18940896 10.1093/humrep/den380

[30] Lin L Tu J Shao J. Acupuncture of different treatment frequency in knee osteoarthritis: a protocol for a pilot randomized clinical trial. Trials. 2019;20:423. 31296249 10.1186/s13063-019-3528-8 PMC6625113

[31] Li S Wang Z Wu H. Electroacupuncture versus sham acupuncture for perimenopausal insomnia: a randomized controlled clinical trial. Nat Sci Sleep. 2020;12:1201–13. 33376432 10.2147/NSS.S282315 PMC7764880

[32] Streitberger K Kleinhenz J. Introducing a placebo needle into acupuncture research. Lancet. 1998;352:364–5. 9717924 10.1016/S0140-6736(97)10471-8

[33] Gao H Lu X Huang H. Thyroid-stimulating hormone level is negatively associated with fertilization rate in patients with polycystic ovary syndrome undergoing in vitro fertilization. Int J Gynaecol Obstet. 2021;155:138–45. 33410141 10.1002/ijgo.13581

[34] Song J Xiang S Yang Y. Assessment of follicular fluid metabolomics of polycystic ovary syndrome in kidney yang deficiency syndrome. Eur J Integr Med. 2019;30:100944.

[35] Debbarh H Louanjli N Aboulmaouahib S. Antioxidant activities and lipid peroxidation status in human follicular fluid: age-dependent change. Zygote. 2021;29:490–4. 33910658 10.1017/S0967199421000241

[36] Pella R Suárez-Cunza S Orihuela P. Oxidative balance in follicular fluid of ART patients of advanced maternal age and blastocyst formation. JBRA Assist Reprod. 2020;24:296–301. 32159933 10.5935/1518-0557.20200012 PMC7365547

[37] Zhang M Lu Y Chen Y. Insufficiency of melatonin in follicular fluid is a reversible cause for advanced maternal age-related aneuploidy in oocytes. Redox Biol. 2020;28:101327. 31526949 10.1016/j.redox.2019.101327 PMC6807363

[38] Kim J Lee H Choi T. Acupuncture for poor ovarian response: a randomized controlled trial. J Clin Med. 2021;10:2182. 34070086 10.3390/jcm10102182 PMC8158119

[39] Lian F Chen C Xiang S. Improvement of the oocyte quality with electroacupuncture in infertility patients of kidney deficiency pattern. Zhongguo Zhen Jiu. 2015;35:109–13. 25854012

[40] Almiñana C Gil M Cuello C. In vitro maturation of porcine oocytes with retinoids improves embryonic development. Reprod Fertil Dev. 2008;20:483–9. 18462610 10.1071/rd07175

[41] Perlmann T. Retinoid metabolism: a balancing act. Nat Genet. 2002;31:7–8. 11953747 10.1038/ng877

[42] Sonneveld E van der Saag P. Metabolism of retinoic acid: implications for development and cancer. Int J Vitam Nutr Res. 1998;68:404–10. 9857269

[43] Pijnappel W Hendriks H Folkers G. The retinoid ligand 4-oxo-retinoic acid is a highly active modulator of positional specification. Nature. 1993;366:340–4. 8247127 10.1038/366340a0

[44] Horton C Maden M. Endogenous distribution of retinoids during normal development and teratogenesis in the mouse embryo. Dev Dyn. 1995;202:312–23. 7780180 10.1002/aja.1002020310

[45] Sonneveld E van den Brink C Tertoolen L. Retinoic acid hydroxylase (CYP26) is a key enzyme in neuronal differentiation of embryonal carcinoma cells. Dev Biol. 1999;213:390–404. 10479456 10.1006/dbio.1999.9381

[46] Wu C Zhao X Wu X. Exogenous glycine and serine promote growth and antifungal activity of Penicillium citrinum W1 from the south-west Indian Ocean. FEMS Microbiol Lett. 2015;362:fnv040. 25761754 10.1093/femsle/fnv040

[47] Brison D Houghton F Falconer D. Identification of viable embryos in IVF by non-invasive measurement of amino acid turnover. Hum Reprod. 2004;19:2319–24. 15298971 10.1093/humrep/deh409

[48] Picton H Elder K Houghton F. Association between amino acid turnover and chromosome aneuploidy during human preimplantation embryo development in vitro. Mol Hum Reprod. 2010;16:557–69. 20571076 10.1093/molehr/gaq040 PMC2907220

[49] Hemmings K Leese H Picton H. Amino acid turnover by bovine oocytes provides an index of oocyte developmental competence in vitro. Biol Reprod. 2012;86:165, 161–112. 22378762 10.1095/biolreprod.111.092585

[50] Xiong J. Effect of Moxibustion at Shenque Point on urine metabolomics of dysmenorrhea model rats. Master. Guangxi University of Chinese Medicine; 2016.

[51] Xia L Zhao X Sun Y. Metabolomic profiling of human follicular fluid from patients with repeated failure of in vitro fertilization using gas chromatography/mass spectrometry. Int J Clin Exp Path. 2014;7:7220–9. 25400819 PMC4230118

[52] Liu L Yin T Chen Y. Follicular dynamics of glycerophospholipid and sphingolipid metabolisms in polycystic ovary syndrome patients. J Steroid Biochem Mol Biol. 2019;185:142–9. 30121347 10.1016/j.jsbmb.2018.08.008

